# Neurophysiological dynamics of visceral signals in emotion, self and bodily consciousness

**DOI:** 10.1098/rspb.2024.2625

**Published:** 2025-06-04

**Authors:** Célia F. Camara, Maria Laura Filippetti, Alejandra Sel

**Affiliations:** ^1^ Department of Psychology, University of Essex, Colchester, UK

**Keywords:** interoception, heartbeat evoked potential, respiratory-related evoked potentials, gastric evoked potentials, electrophysiology, brain–body interaction

## Abstract

Bodily organs such as the heart and the lungs play a crucial role in maintaining physiological homeostasis in a continuous closed-loop interaction with the brain. Beyond their vital role, recent developments have emphasized the remarkable contribution of bodily signals to high-level brain functions. A direct route by which bodily signals influence brain functioning is via modulation of electrophysiological dynamics, which in turn influences the integration and processing of emotional and self-related information regulating our conscious experience. Drawing on electrophysiological investigations, we provide a comprehensive picture of the electrophysiology of interoception and its contribution to emotion, self and bodily consciousness, with a focus on cardiac, respiratory and gastric interoception. We provide evidence that altered neurophysiological responses in interoception might underlie deficits in psychopathology. We also summarize the limited evidence on the development of the electrophysiology of interoception during infancy and adolescence, as well as describing some attempts to investigate causality in the neural mechanisms underpinning interoception. A number of important areas for further research are highlighted.

## 1. Introduction

For decades, the major focus of human science research has been to understand the brain mechanisms underpinning responses to external stimuli, ignoring the fact that brains do not work in isolation but are embedded in a closed-loop interactive brain–body system. Recent developments have emphasized the remarkable ability of the brain to perceive signals emerging from within the body, and to integrate and leverage these signals to guide behaviour. This ability, often known as interoception, provides a moment-to-moment mapping of the body’s internal states [1,2].

Bodily organs continuously send sensory information to the brain; while the research has primarily focused on cardiac interoception due to the measurable nature of heartbeats and the lack of conscious control, interest in other interoceptive domains like respiration and digestion is increasing. However, the mechanisms by which the brain integrates information from these interoceptive domains to guide actions remain largely unknown.

In this article, we highlight and summarize the main findings developed in the investigation of the electrocortical dynamics of interoception in humans, with a focus on cardiac, respiratory and gastric interoception. While these domains differ in some ways (e.g. the heart and the gastrointestinal tract generate their own oscillatory activity, whereas respiratory cycles are generated in the central nervous system), they also share some important features. Thus, all three domains resonate at oscillatory frequencies (from milliseconds to approximate 20 s) matching the time scales of perception and cognition, which provides an important starting point for understanding the impact of interoception on brain and behaviour [3]. Our review focuses on human studies, gathered from peer-reviewed journal articles using databases such as PubMed and Google Scholar. The keywords used in our search included ‘interoception AND emotion’, ‘interoception AND self’, ‘interoception AND consciousness’, ‘interoception AND development’, ‘interoception AND psychopathology’, ‘interoception AND EEG’, ‘interoception AND ERPs’, ‘interoception and tVNS’ and ‘interoception and TMS’.

First, we will provide a summary of the relevant electrophysiological studies that investigated cortical interoceptive responses to the different bodily organs at rest. Second, we will review influential studies highlighting the contribution of interoceptive signals to bodily self-awareness and emotion. Our focus on emotions and bodily self-awareness stems from research and theory evidencing how cardiac and gastric signals are closely tight to one’s own emotional and bodily states, and their conscious experience. Third, we will examine evidence suggesting that altered interoceptive neurophysiological responses might underlie affective and cognitive deficits in psychopathology. Fourth, we will focus on a small number of studies that explore the development of interoception during infancy and adolescence. Fifth, we will summarize research investigating the causality of brain–body dynamics by combining magnetic or electrical stimulation with electrophysiological recordings. Finally, we will outline pressing issues in human interoception research and suggest future directions to advance our knowledge of the neurophysiological mechanism underpinning interoception.

## 2. Mapping the neural pathways from the body to the brain

**Figure 1.**
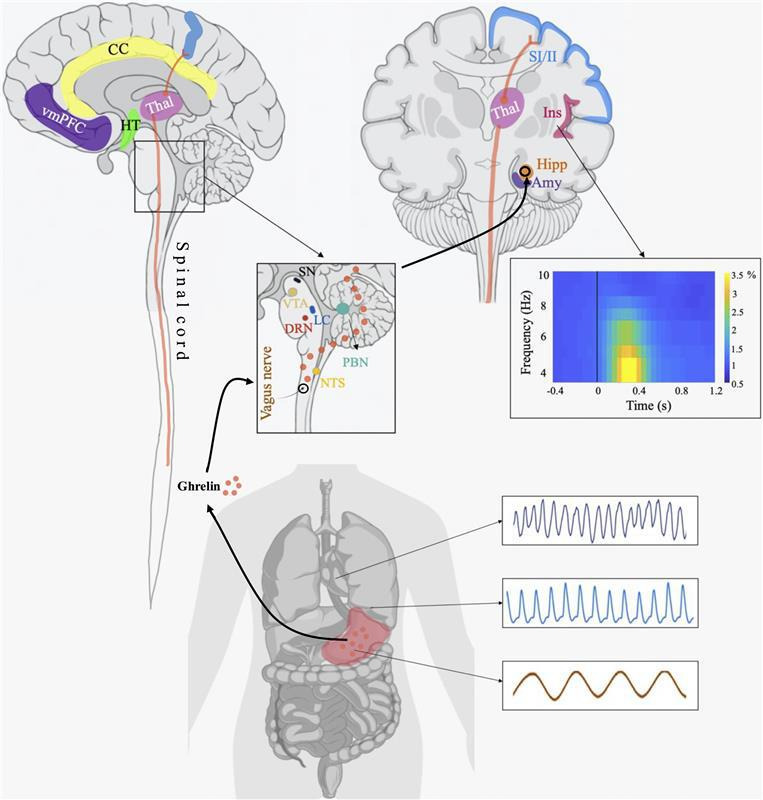
Anatomical structures underpinning interoception. Bodily signals from the heart, lungs and stomach reach the brain through the vagus nerve and spinal nerves. These signals convey information about the internal state of the body. They are processed in subcortical regions like the medulla, pons, nucleus of the solitary tract (NTS) and parabrachial nucleus (PBN). From there, they are transmitted to various brainstem structures, including the dorsal raphe nucleus (DRN), locus coeruleus (LC), substantia nigra (SN) and ventral tegmental area (VTA), as well as other subcortical regions like the thalamus (Thal), hypothalamus (HT), hippocampus (Hipp) and amygdala (Amy). Parabrachial projections to the thalamus eventually reach cortical areas such as the primary and secondary motor cortices (SI/II), insula (Ins), ventromedial prefrontal cortex (vmPFC) and cingulate cortex (CC)—an example of a spino-thalamic pathway is illustrated in orange. Additionally, bodily information can travel through the endocrine system, where hormones from organs like the stomach (e.g. ghrelin) enter the bloodstream to reach target tissues, including the brain. The graph on the right shows an EEG time–frequency representation recorded during a task, which simulates electrophysiological activity time-locked to the heart. Cortical and body images created with BioRender (https://biorender.com/)

Bodily organs provide signals about the internal state of the body through mechanical and chemical sensors. These signals reach the brainstem nuclei via the vagus nerve and the spinal splanchnic nerve, and this information is then sent to other structures like the thalamus, amygdala, hippocampus, insula, cingulate cortex or sensory cortex (figure 1), which are associated with key cognitive and emotional processes [4]. Additionally, bodily information can travel via the endocrine system pathways where hormones conveying information about an organ travel in the bloodstream towards the targeted tissues, including the brain. Another interoceptive pathway is the humoral immune system, which transmits information to the brain about peripheral states of infection and inflammation [5], resulting in automatic responses ranging from cardiovascular reflexes to social isolation and anhedonia [6].

Electrophysiological studies of interoception involve co-registering cortical and autonomic activity, followed by offline examination of the electrocortical events time-locked to changes in autonomic activity. Polarity changes that occur in the ventricular tissue leading to the QRS complex of the heartbeat are accompanied by an evoked electrophysiological response in the brain; a deflection around 200−400 ms after the R-peak onset. This event-related potential (ERP), known as the heartbeat evoked potential (HEP) or the heartbeat evoked response (HER) [7,8], is the most used neurophysiological marker of cardioception. The properties of the HEP have been reviewed thoroughly elsewhere [8,9]; thus, we will focus on the neurophysiological properties of two other interoceptive domains: respiration and digestion.

Like cardiac interoception, the brain shows cortical responses associated with breathing. Most studies have examined resting brain activity or during breathing manipulations like occlusions. However, few have explored cortical responses to breathing during emotional/cognitive tasks. We will focus on research involving natural breathing and techniques like threshold loading or breathing occlusion, reviewing the brain-body pathways that generate cortical responses and highlighting key electrophysiological findings in human studies.

Intracranial EEG (iEEG) studies in humans show that natural breathing synchronizes activity in the olfactory cortex and limbic areas, including the amygdala and hippocampus. This suggests that respiratory rhythms modulate oscillatory responses in regions associated with complex behaviours and orofacial sensations [10]. However, iEEG is spatially limited, potentially missing oscillatory changes in other areas synchronized with the breathing cycle.

Similarly to the lungs, the stomach exhibits a continuous gastric basal rhythm at infra-slow frequency (approx. 0.05 Hz; 3 cycles per minute). The gastric rhythm is continuously generated regardless of whether digestion is taking place, and even when the stomach is disconnected from the central nervous system [11]. The stomach cells connect to vagal afferent sensory neurons [12], which send information via the thalamus to various cortical structures within the visceral brain circuit, including the somatosensory cortex, the ventromedial prefrontal cortex (vmPFC) and the cingulate motor areas (see [13] for a review). A recent study found that the phase of infra-slow gastric activity is coupled with alpha oscillatory power in the parieto-occipital sulcus, calcarine fissure bilaterally and the right anterior insula (rAI). This coupling appears to be driven by ascending signals from the stomach, indicating that the gastric basal rhythm originates in the stomach and may help orchestrate large-scale brain activity dynamics at rest [14].

Beyond techniques such as the water loading task (where participants drink a set amount of water while monitoring their brain activation [15]), recent approaches use a minimally invasive protocol where participants ingest small vibrating capsules that produce gastric sensations they ought to report. The perception of vibrations was linked to a ‘gastric evoked potential’, a late positive ERP over parieto-occipital regions between 300 and 600 ms after the vibration onset [16] (see [17,18] for a similar approach).

## 3. The role of visceral cortical responses on emotion and bodily self-awareness

Emotional experience and bodily self-awareness represent two important functions of interoception. These two functions are intricately connected as historically bodily signals have been associated with emotions [19]. Recent theories suggest that interoceptive and exteroceptive signals are integrated in the brain to anticipate homeostatic control (i.e. allostasis [20–22]). These theories are described within the predictive processing (PP) perspective [23] suggesting that emotions are shaped by the brain’s ability to anticipate and interpret bodily signals, forming a dynamic relationship between emotional states and awareness of bodily sensations [24,25]. Predictions rely on past experiences (priors) and are updated based on the sensory input; any mismatch generates prediction errors that must be resolved by adjusting the internal model or altering the sensory data [24–26]. The active inference-based view of Barrett *et al.* [20,21] propose that affective experiences involve constant monitoring, adjusting and updating of interoceptive predictions and bodily states to keep bodily states within an expected range. Emotions arise by integrating information from different interoceptive and exteroceptive channels that form interoceptive predictions, which are then compared to incoming signals.

Since interoceptive information reflects one’s bodily states, it is unsurprising that the PP coding framework now includes bodily self-awareness [2,27–29]. Own-body representations are thought to integrate predictions about the body with exteroceptive, proprioceptive, vestibular and visceral signals. Evidence linking interoceptive abilities and self-awareness [30–34] suggests that, while exteroceptive models shape body representations, interoception provides stability against external changes [35]. Visceral signals, especially continuous signals like cardiac and gastric inputs, are proposed to offer a bodily-centred frame of reference, coordinating sensory and cognitive brain maps [28,29].

Yet it is also important to acknowledge that bodily self-awareness is not necessarily always tied to an emotional experience. For example, bodily self-awareness results from the integration of exteroceptive and interoceptive signals [36], as evidenced by experiments employing bodily illusions (see [35] for a review). Such bodily illusions can be triggered by congruent or incongruent visual and tactile (exteroceptive) stimuli [37], or by the integration of exteroceptive and interoceptive signals like visuo-cardiac or visuo-respiratory cues [32–34,38,39].

### (a) Cardiac interoceptive contribution to emotion and self-awareness

Most of the neurophysiological studies on visceral brain responses and bodily self-awareness/emotion in humans focus on cardiac interoception, particularly using the HEP. Specifically, participants perform tasks involving external information (e.g. emotional expressions, self-images) while their HEP is recorded with EEG. Amplitude HEP changes recorded during the task are a proxy of cardiac interoceptive contribution to emotion and self-processing. As previous work gives a detailed overview of studies investigating cardiac interoception with HEP in emotion and self-awareness [8,19,40], we provide a critical summary of recent key developments on cardiac electrophysiological responses in these domains.

The observation of emotional stimuli—facial expressions, affective pictures or emotional movie clips—leads to a negative amplitude modulation (250–550 ms after the R-peak) over fronto-central electrodes [41–43]. One study investigated the neural sources of these HEP changes in response to sadness, revealing a network involving the pre-frontal cortices, the globus pallidus, the rAI and the anterior cingulate cortex (ACC). They demonstrated increased information flow from the rAI to the right ACC when observing sad expressions, suggesting an integration of cardiac signals during emotional processing [42]. Moreover, expectations about upcoming emotional information influence cardiac neural processing [44,45]. Likewise, HEP amplitudes are modulated when thinking about past/future affective experiences [46] and when perceiving high (versus low) arousing audio-visual stimulation [47]; also, listening to sounds of disgust modulates beta oscillations and connectivity between the insula and pre-frontal cortex [48].

Electrophysiological studies investigating interoception in self and body awareness show that watching a picture depicting one’s face morphed with the face of another that pulses in synchrony with one’s heartbeat leads to a positive amplitude modulation of the HEP, accompanied by an increased self-identification with the other’s face [33]. The magnitude of the HEP modulation relates to the individuals’ abilities to sense their heartbeat, known as interoceptive accuracy, measured by the heartbeat counting task [49]. Similarly, changes in body-ownership manipulated with the full-body illusion show HEP amplitude modulations [50] linked to fluctuations in insular and posterior cingular activity [50,51]. Moreover, directed thoughts and mind-wandering focused on self-related versus non-self-related content result in HEP amplitude changes originating in the precuneus, vmPFC, mid-posterior cingulate regions and insula [52,53]. Interestingly, resting-state HEP can differentiate conscious from unconscious states in post-comatose patients [54], and during a task it can also reflect self–other emotional distinction [55].

Some investigations on cardiac interoception and emotion/self-awareness report HEP amplitude changes across varying time windows and scalp topographies. This variability, often related to inconsistent methodologies (see [40] for a full review) limits clear conclusions about interoception’s role in emotion and consciousness. On the other hand, high-arousing information is known to heighten autonomic responses (e.g. increased heart rate [56]), suggesting that HEP amplitude may reflect autonomic rather than interoceptive responses [19,40]. Furthermore, the role of cardiac interoception in emotional and self-awareness states driven by non-visual sensory information remains poorly understood and warrants further investigation.

### (b) Respiratory interoception and its role in self-awareness and emotion processing

Respiratory pace and depth are associated with changes in bodily arousal, like those experienced during anxiety [57] and stress [58], and this relationship is bidirectional. At rest, respiration orchestrates oscillatory and non-oscillatory activity across all EEG frequency bands throughout a widespread brain network [59,60]. Similarly, the respiratory cycle can influence neural excitability during a perceptual task whereby perceptual sensitivity and neural excitability—measured by pre-stimulus alpha oscillatory changes—are shown to be enhanced at a respiration phase lag of approximately −30° [61]. Moreover, recent evidence suggests that during exhalation, the HEP amplitude is increased as opposed to exhalation at rest [62], showing some initial evidence of the integration of the two interoceptive modalities in the brain.

Other studies focused on respiratory-related evoked potentials (RREPs) recorded from EEG to investigate how respiratory interoception varies in response to affective stimuli. RREPs are measures of cerebral cortical activity elicited by occluding respiration short after inspiration onset [63]. RREPs studies suggest that respiratory interoception is modulated by affective responses. Von Leupoldt *et al*. [64] observed reduced P3 magnitude and enhanced late positive potentials (LPP) to occlude respiration during the observation of affective (pleasant, unpleasant) versus neutral stimuli in healthy individuals. They also found the occlusion to be felt less pleasant when viewing unpleasant pictures.

Noteworthy, the procedure involving respiration occlusion can be a negative experience itself, leading to confounding results on the impact of respiration in emotion processing. Alternatively, breath-locked EEG can probe how respiratory interoception varies with and influences affective processing.

### (c) Gastric interoception is linked to affective processing

High-arousal negative states, like stress, are thought to alter gastric rhythms via brain-to-stomach efferent signals [13,65]. Some initial evidence suggests that the gut-brain relationship is complex and bidirectional, and that gastric information is likely to have multiple effects on cognition, affection and motivation. For example, gastric interoception has been linked to the AI and orbitofrontal cortex (regions involved in affective and cognitive processing [66]) and to the amygdala and hippocampus, which help recreate past gut sensations [67].

The brain–gut relationship has been widely studied in chronic gastrointestinal and eating disorders. Nomura *et al*. [68] evaluated the gut–brain interaction during stress induced by an arithmetic test in healthy people and patients with irritable bowel syndrome (IBS). Compared with healthy participants, IBS patients exhibited a significant increase in beta power, which positively correlated with colonic motility [68]. Further evidence shows that viewing angry words increases rectal tone, particularly in IBS patients, and this is linked to enhanced N400 response (cortical index of semantic processing [69]). Relatedly, reducing disgust-related gastric dysrhythmias with a drug decreases oculomotor disgust avoidance [70]. These findings indicate that gut motility could serve as a dynamic indicator of emotion processing, although it is possible that this relationship is mediated by other factors, as indicated by the differences between IBS and healthy controls [69].

A common technique used to investigate the linkage between affection and the gut is the water loading technique, specifically designed to measure gastric distension and trigger the mechanical receptors in the stomach [71]. Using this technique, van Dyck *et al*. [72] found that an increased sensation of water fullness elicits more negative affect in both healthy participants and patients with eating disorders. Information from the gut is not only encoded by the mechanical receptors but also by immune cells and by enteroendocrine cells. Thus, another route in the gut–brain axis that has gained increased attention refers to interoceptive inputs from intestinal microbes, proven to have an influence on emotional arousal and affective behaviour, including negative feelings of hunger and satiation, and positive feelings derived from food flavors [67].

Finally, an interesting view from Vianna *et al*. [73] hypothesizes that digestive signals—specifically those from the gut—may play a more prominent role in shaping the overall experience of emotion when the emotional experience is not vividly felt. This hypothesis suggests that changes in the digestive system, such as increased gastric rhythm and altered gut motility, could in turn modulate brain dynamics influencing cognition and emotion [74]. In this line, recent evidence links more acidic stomach pH to greater self-reported feelings of disgust, and less acidic pH to reported happiness [18]. Future investigation of gastric interoception during both affective and cognitive tasks could shed light on this possibility.

## 4. Altered visceral cortical responses in psychopathology

Atypical interoception is relevant for psychopathology, with increasing evidence linking deficits in interoception—particularly cardiac interoception—to mental health disorders [75–78]. Theories propose that impaired neural processing of interoceptive signals can disrupt the central system responsible for maintaining physiological homeostasis [21]. When this system fails to adaptatively respond to the bodily demands or generate predictions based on past bodily states, the organism might reach allostatic overload (i.e. excessive ‘wear and tear’ of the body), resulting in psychopathology [77,78]. According to untested hypotheses, this allostatic overload might be the underlying cause of commonality in symptoms across several mental health disorders including anxiety, depression, obsessive compulsive disorder (OCD) and even disorders with a high psychotic component such as borderline personality disorder. Here, we examine studies linking altered electrocortical interoceptive responses and psychopathology.

### (a) The involvement of cortical cardiac processing in psychopathology and mental health

Numerous studies have explored the link between altered electrocortical responses to cardiac signals and psychopathology. One pioneering investigation in this field, conducted by Terhaar *et al*. [79], focuses on depressed patients. In this study, they found a significant reduction of the HEP amplitude in depressed individuals in comparison with healthy controls, suggesting that the HEP could serve as a neural marker of altered bodily awareness according to the somatic marker hypothesis [80]. These results are consistent with behavioural studies showing that depression is linked to impaired interoceptive accuracy [81,82], as well as with neurofunctional evidence indicating that depressed individuals exhibit decreased mid, dorsal and posterior insula activity when attending to the heart, as well as during recollection of negatively conditioned stimuli [83–85].

Likewise, evidence of alterations in HEP amplitude can be found for anxiety. For example, individuals with high social anxiety (compared with controls) show an increased HEP amplitude when receiving false feedback of increased heart rate, and these HEP changes were associated with reported anxiety symptoms [86]. Moreover, high levels of stress lead to a negative increase of HEP amplitude over left temporal and lateral pre-frontal areas [87–90]. This HEP amplitude modulation has been associated with enhanced self-focus driven by concerns that features of the self, such as external validation cues, may cause anxiety [86,91,92]. In this line, individuals with generalized anxiety show a significant reduction of grey matter volume in the ACC, a primary interoceptive area [91].

Furthermore, altered cortical interoceptive responses have also been observed in OCD, borderline personality disorder and depersonalization disorder. For example, individuals with OCD exhibit greater HEP amplitudes during an interoceptive task involving tapping one’s finger along with one’s versus others’ heartbeats, while showing a decrease in confidence and awareness of their interoceptive skills [93]. In the same vein, borderline personality disorder is associated with both amplitude increased and decreased HEP amplitudes in frontal and parietal sites, along with higher heartrate variability [94] and emotional dysregulation [95]. On the other hand, patients with depersonalization disorder show no difference in HEP amplitude between rest and a heartbeat counting task [96,97].

Overall, mental disorders have been linked to an altered processing of cardiac interoception characterized by HEP amplitude increases (except for depression, where HEP decreases are observed), and HEP amplitude modulation occurs concomitantly with deficits in interoception at the behavioural level—such as decreased interoceptive accuracy and awareness. The mismatch between neural and behavioural responses observed across a range of mental health issues has been proposed as a key factor to explain allostatic overload that leads to psychopathology [98]. Whether the interoceptive deficits originate in the periphery (e.g. sensory receptors, vagus nerve transmission path) or at the central level in the brain areas where bodily signals are integrated is still an open question.

### (b) Respiratory and gastric interoception in relation to mental health

Contemporary theories argue that inaccurate interoceptive signals can cause individuals to rely more heavily on expectations, which in turn can alter their perception of breathlessness and exacerbate the mismatch between subjective and objective bodily responses [99]. This is particularly relevant in the context of anxiety, where respiratory sensations that would otherwise be ignored may become amplified, leading to increased symptoms of breathlessness and potentially resulting in faulty interoceptive inferences [99]. Interestingly, higher alertness to respiratory sensations in anxious people seems related to a decreased sensitivity to stimuli of respiratory resistance [100]. In line with this, evidence from RREP research has shown that increased anxiety is associated with both higher cognitive perception of breathing [101,102] and reduced respiratory sensory gating [103,104].

Respiration disturbances are very common across a plethora of psychiatric conditions (e.g. anxiety, depression, OCD [105]). Problems in the respiratory system can significantly affect cognition and brain function, exacerbating the symptoms of psychopathology. Studies have found that dyspnoea (or shortness of breath) is linked to enhanced feelings of unpleasantness and arousal [106,107], and that the unexpected occurrence of dyspnoea results in increased anxiety during cognitive processing [108].

The current work seems to suggest a bidirectional relationship between respiratory interoception and psychopathology. However, this research has so far been limited to anxiety disorders. Considering the commonality of respiratory symptoms across many other mental disorders, studies on other prevalent conditions such as depression [109] are needed.

Contemporary research suggests that a dysfunctional communication pathway between the gut and the brain can impede active interoceptive inference [20,110]. For example, unpleasant gastrointestinal changes may lead to inaccurate interoceptive inferences that can then result in a temporary physiological change such as inflammation [111]. If repeated, these episodes increase the incongruence between incoming sensory signals and a falsely predicted sensory cause [110], thereby heightening anxiety and contributing to visceral hypersensitivity. This heightened sensitivity to visceral sensations, induced by negative emotions, can in turn increase psychological stress and aggravate affective disturbances—such as anxiety or depression—in a feedback loop manner [28].

This circular relationship is reflected by the high comorbidity between mood disorders and gastrointestinal diseases [112]. Thus, for example, the mismatch between objective interoception and generated concept responses is linked to activity in the insula, the ACC and the pre-frontal cortex [67]. Patients with comorbid IBS and anxiety/depression present abnormal activity in the dorsolateral PFC and the ACC [113]. This provides further support for the role of interoceptive-related neural processing associated with visceral sensation in the emergence of psychopathology.

Noteworthy, neuroimaging studies in individuals with psychopathology have revealed altered activations in areas of the interoceptive circuit (e.g. the ACC), there is currently no evidence linking altered electrocortical responses and altered gastrointestinal rhythms. Therefore, further extensive research is needed to investigate the electrocortical mechanisms underlying gastric interoception and their potential relation with psychopathology.

## 5. The development of visceral brain responses

If interoceptive signals are a central feature in perception, cognition, emotion and consciousness, then understanding when and how interoceptive processing emerges in early life becomes imperative. Recent frameworks emphasize the intersubjective developmental nature of interoception, highlighting the importance of carer–infant interactions for energy expenditure, temperature and immune function [114,115]. Accordingly, early carer–infant interactions can influence infants’ ability to identify specific interoceptive changes, impacting their behavioural responses for allostatic regulation [116]. Notwithstanding the fact that carers ultimately determine whether the infants’ needs are acknowledged, other accounts have also acknowledged infants’ ability to register physiological changes and actively communicate them [117] before any meaning can be attached to the sensations experienced [118]. In this section, we focus on the existing literature on cardiac, respiratory and gastric interoception in this context.

### (a) The developmental study of cardiac interoception

Only a few studies have measured the cardiac brain responses throughout the development, focusing on infants [119–121] and adolescents [122]. Maister *et al*. [119] measured cardiac brain responses of 5 month infants while viewing short video clips of emotional and non-emotional facial expressions. These studies showed an HEP amplitude enhancement in frontal electrodes in the 150−300 ms time window after the R-wave when infants observed video clips with fearful and angry expressions in comparison with neutral expressions. This HEP amplitude difference was particularly obvious in those infants who were better able to discriminate between an animated character that moved either in synchrony or asynchrony (±10% speed) with the infant’s own heartbeat, therefore suggesting greater implicit sensitivity to their own cardiac interoceptive signals [119]. This pioneering study opens the way for further investigations of electrophysiological responses to interoceptive cardiac signals, and signals from other interoceptive domains, in infants, children and adolescents (e.g. [122]).

### (b) Respiratory and gastric interoception in development

Despite the limited research on the development of respiratory interoception (although see recent work by Tünte *et al.* [120]), some studies show that respiration has an impact on children’s development. A programme developed for children in Korea revealed that respiration training increases brain functioning related to important cognitive functions like memory and creativity [123]. The programme included physical/mental exercises alongside training interoceptive awareness via guided respiration. This training led to alpha increases in left frontal regions and beta decreases [123], previously linked to emotional maturation in children [124]. Additionally, clinical studies show that children with sleep apnoea—who rely more on mouth breathing—have lower educational achievement than those who primarily use nasal breathing [125,126].

Moreover, paediatric studies demonstrate that breathing disorders impact cardiac interoception highlighting how one interoceptive modality can affect another during development. Thus, children with sleep disorder breathing, as opposed to healthy controls, exhibit decreased HEP amplitude (400−550 ms after R-peak) during expiration versus inspiration in REM sleep [127,128].

Neurodevelopmental studies have shown that the development of the brain and the gastrointestinal tract occur in parallel during the first years of life [129]. Some proposals argue that newborns are able to form basic representations of their emotional states via afferent signalling from the gut in a homeostatic process that helps the infant to adapt to their environment [129], and that this process heavily relies on early dyadic interactions with their carers [116,130]. Thus, for example, early disturbances in the gastrointestinal system such as paediatric IBS can indirectly influence cognitive functioning by hindering the infant’s capacity to adapt to the environment. Moreover, evidence from gut microbiota studies shows that lower efficiency of the gut microbiota in prenatal infants is linked to greater risks for brain inflammation and neurodevelopmental disorders [129], which can impact brain functioning and cognition.

To our knowledge, direct evidence of developmental changes in electrophysiological brain responses to respiration and/or gastric interoception and their impact on cognition is lacking. Therefore, it is crucial for future research to explore the neurophysiological markers of respiratory and gastric interoception, and their relationship with cognition during development.

## 6. Manipulation of brain and body visceral responses

Our current understanding of the neurobiology of interoception builds on correlational neuroimaging studies, mainly EEG and fMRI and some lesion studies. However, lesions often spread over multiple neighboring regions and therefore it is difficult to delineate the direct contribution of a specific brain region to interoceptive processes.

Advancements in neurostimulation techniques have facilitated the investigation of the causal role of specific brain regions in neural processes and associated behaviours. Transcranial magnetic stimulation (TMS)—and other forms of brain stimulation such as electrical stimulation (e.g. transcranial alternating current stimulation)—are often used to investigate causality and directionality within a given cortical network. The interoceptive neural network comprises both cortical and subcortical regions. While it is possible to investigate the causal role of interoceptive cortical regions such as the somatosensory cortex with neurostimulation, deeper brain areas such as the insula or the amygdala are difficult to reach with traditional neurostimulation methods.

As neuroimaging evidence accumulates, it is becoming clear that the continuous, rhythmic fluctuations of bodily responses do not only trigger an event-related brain response (e.g. HEP, RREP) but also influence both resting activity and task-related brain activity in several cortical regions such as the visual, auditory and sensory regions. There is substantial evidence demonstrating that both the cardiac and the respiratory rhythms directly affect high-level cognitive processes, such as perceptual awareness [131,132]. However, the impact of bodily rhythms on brain function extends beyond the scope of the current review and warrants a separate review (see [133]).

### (a) Causal interventions in cardiac interoception

Thus far, two studies from the same research group have explored the causal role of known interoceptive structures in cardiac interoceptive processing with a common neurostimulation protocol: continuous theta-burst stimulation—cTBS [134,135]. Pollatos *et al.* [135] applied cTBS placing the TMS coil over the right somatosensory cortex and frontotemporal regions aiming at inhibiting the right somatosensory cortex and the right insula. After the cTBS protocol, participants exhibited reduced HEP amplitudes over frontal sites alongside increases in confidence rates in the heartbeat counting task [135].

Although cTBS is viewed as an effective method for examining causal relationships in areas within the interoceptive network [135], criticisms regarding the methodology and results remain [136,137]. A primary concern is the difficulty in reaching the deep cortical region of right insula using Pollatos *et al*.’s parameters and protocol [127], leaving uncertainty as to whether observed effects were due to direct stimulation of right insula, indirect stimulation of connected areas or activation of interoceptive regions within the ventromedial pre-frontal cortex [130,131]. Furthermore, using the 10/20 EEG system to establish stimulation coordinates often introduces inaccuracies, reducing reliability. The absence of a control condition in these studies further raises questions about whether observed impairments in the interoceptive task were specific to interoception or indicative of general cognitive deficits [128].

Another way of studying the causal influence of bodily signals on brain processing is by manipulating bodily afferences before they reach the central nervous system. Electrical stimulation techniques, like transcutaneous vagus nerve stimulation (tVNS), provide a non-invasive method to investigate the role of peripheral bodily signals in brain and cognitive processing [138–143]. While the exact mechanism underpinning the tVNS effects on the vagus nerve are yet unknown, active tVNS on the left vagus nerve (20−30 Hz; 0.25−3.5 mA; 30−60 s ‘on’ and 5 min ‘off’) [144] modulates noradrenaline levels, leading to increased neural excitability [145] alongside increases in theta and alpha power, and decreases in beta and gamma [140,146].

Moreover, tVNS is shown to alter the HEP amplitude [137,141]. Richter *et al*. asked participants to engage in an interoception task, tapping on a keyboard in sync with their heartbeats, alongside a control task involving tapping to a sound. The tVNS group exhibited enhanced HEP amplitudes and improved interoceptive performance compared with the sham group, with no changes noted in the exteroceptive condition [141]. Similarly, tVNS modulates activity within interoceptive neural network, linked to increased P1–N1 amplitudes [139]. Additionally, tVNS induces transient pupil dilation and attenuates occipital alpha oscillations, both markers of arousal [140].

The findings suggest that tVNS can modulate the cortical state of the interoceptive brain circuit, offering electrophysiological evidence for vagal signals in processing bodily signals. These results may help identify biomarkers of bodily allostasis in healthy and diseased populations, with clinical implications that warrant further exploration.

### (b) Causal interventions in other interoceptive domains

Brain stimulation techniques have not yet been applied in basic research to explore the neural mechanisms of respiration and gastric interoception and their connection to cognition. Most studies on the effects of brain stimulation in these areas have been conducted in clinical settings. For instance, deep brain stimulation has significantly enhanced lung function, particularly when targeting the periaqueductal grey matter and the subthalamic nucleus [147]. Stimulating vagal afferent gut fibres is used to alleviate symptoms of psychiatric disorders like anxiety and depression by reducing inflammation and regulating key neurotransmitters, such as serotonin, which is crucial for appetite regulation [148].

The observed changes in the interoceptive cortical network after manipulating cortical excitability or active vagal modulation warrant further investigation. However, a key limitation of these studies is the potential risk of physiological dysfunction in healthy individuals when applying brain stimulation to areas controlling respiratory or cardiac signals. Additionally, some critical regions, like the amygdala and insula, are not on the cortical surface and cannot be easily targeted using standard TMS methods. Therefore, future studies must address these limitations by developing and optimizing new forms of neurostimulation, such as the pioneering focused ultrasound stimulation technique, which allows targeting of subcortical interoceptive regions in a non-invasive manner. Furthermore, future research should also aim to explore the potential of innovative neurostimulation techniques for more precise and controlled manipulation of interoceptive processing in deep brain structures.

## 7. Final remarks and future directions

This article provides a comprehensive review of the current evidence on brain electrophysiological responses to interoceptive bodily signals. We focused the review on three interoceptive domains (i.e. cardiac, respiratory and gastric interoception) that share common pathways and whose influence on emotion and bodily self-awareness has been proposed by recent theoretical frameworks and evidenced by some research studies. Nevertheless, there are still important gaps in our knowledge of how brain–body signals are integrated and may influence perception and cognition. Importantly, experimental studies specifically (and explicitly) targeted at testing candidate mechanisms proposed by theoretical accounts are necessary to provide support to these hypotheses. In this review, we suggest that using neurophysiological indices of interoception can be instrumental in shedding light into the functional role of interoception.

This review highlights that, although cardiac interoception’s role in emotion, self and consciousness is well supported, there is limited knowledge on the brain’s responses to respiratory and gastric signals and their effects on cognition and behaviour. Future research should develop multimodal approaches to explore how the brain integrates signals from respiration, digestion and other domains.

While consistent evidence links brain responses to the heart, especially the HEP, to various affective and cognitive processes, there are significant limitations in studying cardiac interoception and its neural mechanisms. A key limitation is the correlational nature of the findings, which makes it challenging to determine whether changes in HEP amplitude during perceptual tasks are simply coincidental with higher cognitive processes, like perceptual awareness. Future research should utilize high-precision brain stimulation techniques to reliably target HEP neural generators, helping to clarify the causal role of HEP in emotion and self-processing. Additionally, advanced analysis methods, including trial-to-trial EEG analysis and machine learning, could enhance the study of interoceptive neurophysiology [7]. Overcoming these methodological changes will significantly advance our understanding of how interoception contributes to our ability to distinguish ourselves from others and to correctly infer emotional states from others, which are key abilities for social cognition.

Understanding the contribution of electrocortical interoceptive signals to mental health is crucial due to their profound impact on individuals’ lives. Investigating alerted interoceptive cortical processing can help tailor interventions that alleviate mental health burdens, improve health outcomes and reduce reliance on medical care. Additionally, the developmental study of brain responses to interoceptive signals is an unexplored area that requires immediate attention. Efforts should focus on how interoception develops across the lifespan. Given its significant implications in psychopathology, future research will help identify individuals with poor interoceptive responses, serving as a valuable tool for detecting vulnerabilities to affective, physical and mental dysfunctions.

## Data Availability

This article has no additional data.
